# *Mycobacterium abscessus* Induces a Limited Pattern of Neutrophil Activation That Promotes Pathogen Survival

**DOI:** 10.1371/journal.pone.0057402

**Published:** 2013-02-25

**Authors:** Kenneth C. Malcolm, E. Michelle Nichols, Silvia M. Caceres, Jennifer E. Kret, Stacey L. Martiniano, Scott D. Sagel, Edward D. Chan, Lindsay Caverly, George M. Solomon, Paul Reynolds, Donna L. Bratton, Jennifer L. Taylor-Cousar, David P. Nichols, Milene T. Saavedra, Jerry A. Nick

**Affiliations:** 1 Department of Medicine, National Jewish Health, Denver, Colorado, United States of America; 2 Department of Pediatrics, National Jewish Health, Denver, Colorado, United States of America; 3 Department of Medicine, University of Colorado Denver, Aurora, Colorado, United States of America; 4 Department of Pediatrics, University of Colorado Denver, Aurora, Colorado, United States of America; 5 Department of Pediatrics, Children’s Hospital Colorado, Aurora, Colorado, United States of America; 6 Denver Veterans Affair Medical Center, Denver, Colorado, United States of America; 7 Integrated Department of Immunology, National Jewish Health and University of Colorado, Denver, Colorado, United States of America; Hopital Raymond Poincare - Universite Versailles St. Quentin, France

## Abstract

*Mycobacterium abscessus* is a rapidly growing mycobacterium increasingly detected in the neutrophil-rich environment of inflamed tissues, including the cystic fibrosis airway. Studies of the immune reaction to *M. abscessus* have focused primarily on macrophages and epithelial cells, but little is known regarding the neutrophil response despite the predominantly neutrophillic inflammation typical of these infections. In the current study, human neutrophils released less superoxide anion in response to *M. abscessus* than to *Staphylococcus aureus*, a pathogen that shares common sites of infection. Exposure to *M. abscessus* induced neutrophil-specific chemokine and proinflammatory cytokine genes. Although secretion of these protein products was confirmed, the quantity of cytokines released, and both the number and level of gene induction, was reduced compared to *S. aureus*. Neutrophils mediated killing of *M. abscessus*, but phagocytosis was reduced when compared to *S. aureus*, and extracellular DNA was detected in response to both bacteria, consistent with extracellular trap formation. In addition, *M. abscessus* did not alter cell death compared to unstimulated cells, while *S. aureus* enhanced necrosis and inhibited apoptosis. However, neutrophils augment *M. abscessus* biofilm formation. The response of neutrophils to *M. abscessus* suggests that the mycobacterium exploits neutrophil-rich settings to promote its survival and that the overall neutrophil response was reduced compared to *S. aureus*. These studies add to our understanding of *M. abscessus* virulence and suggest potential targets of therapy.

## Introduction

The rapidly growing mycobacteria, *Mycobacterium abscessus,* was first isolated from a chronic suppurative leg infection, and the species was named based on its association with deep abscesses in human tissues [1]. *M. abscessus* is now understood to be an opportunistic pathogen, present in the environment and within water distribution systems [2], [3]. Common clinical scenarios in which this organism occurs include skin and soft tissue infections following trauma, needle sticks, or surgical procedures in patients with no pre-existing immunodeficiency [1], [4]–[7]. It is also isolated in the respiratory tract of patients with cystic fibrosis (CF) and non-CF bronchiectasis, conditions that feature obstruction of the bronchi by mucous plugs, chronic infections, and a purulent milieu of dead and dying neutrophils [5], [8]–[13]. The profile of infection by *M. abscessus* mirrors in many respects that of a common CF pathogen, *Staphylococcus aureus*.

To date the initial host response to *M. abscessus* has been studied in macrophages, epithelial cells and endothelial cells [14]–[16]. Given the abundance of neutrophils in *M. abscessus*-infected tissues [4], we believe a critical element in understanding pathogenesis of *M. abscessus* infection is the association between *M. abscessus* and neutrophils. Massive quantities of polymorphonuclear cells are the most consistent finding within pathologic specimens of cutaneous *M. abscessus* infection [4], and in the CF airway. Opportunistic growth of *M. abscessus* in neutrophil-rich environments suggests that not only is *M. abscessus* capable of thwarting the bactericidal capacity of neutrophils, it may also promote an intense pro-inflammatory milieu within an abscess. These conditions may provide a survival advantage for the pathogen. To our knowledge, this aspect of the host response to *M. abscessus* has not been investigated.

The increased clinical prevalence of *M. abscessus* is concerning both because of its virulence and natural resistance to antibiotic treatment [17]. The organism is capable of forming a biofilm [18], a virulence mechanism common to other chronic infections of soft tissue and the CF airway, which evokes further antibiotic resistance, immunoresistance, and persistent infections [17]. A second identified characteristic of *M. abscessus* is the absence or presence of a glycopeptidolipid (GPL) on the cell surface; GPL is expressed on the “smooth” colony morphotype, whereas the “rough” colony morphotype lacks GPL expression [19]. Smooth *M. abscessus* appears to be the predominant morphotype in the environment, and is most frequently associated with initial infections [20], but also persists during chronic infection [20], [21]. Smooth strains are more successful biofilm producers, suggesting that the smooth variant establishes a biofilm foothold [14], [17].

We tested well-defined neutrophil responses to *M. abscessus*, and compared the responses to those of *S. aureus*, a well-characterized CF pathogen that is also commonly implicated in abscess formation. In all cases, the response to *M. abscessus* was blunted when compared to that of *S. aureus*, suggesting that one survival advantage of *M. abscessus* is reduced immune response. Furthermore, in the presence of dead and dying neutrophils the capacity of *M. abscessus* to form a biofilm was dramatically enhanced. Together, our data suggests that *M. abscessus* exploits neutrophils by eliciting a limited and dysregulated immune response, as well as accelerating biofilm formation, which may promote persistence of infection.

## Methods

### Bacterial Strains, Media and Culture Conditions

*M. abscessus* (ATCC strain 19977) was propagated in 7H9 broth supplemented with 0.5 g/l bovine albumin fraction V, 0.2 g/l dextrose, 0.3 mg/l catalase (ADC; BD Biosciences), 2% glycerol and 0.05% Tween-80 at 37°C with shaking at 300 rpm for 3–5 days. Plating of the broth culture resulted in two distinct colony phenotypes, one smooth in appearance and one rough. These colony types were isolated and propagated separately in 7H9 broth with ADC and maintained as separate rough and smooth strains; the smooth variant was used throughout this study. GPL production was confirmed for smooth *M. abscessus* using thin-layer chromatography [18]. DNA analysis confirmed the species as *M. abscessus* (rather than *M. massiliense* or *M. bolletii*) [22]. Aliquots of cultures at a density of McFarland 1 were stored at −80°C after day 5 of culture. For assays, bacteria were thawed at room temperature and grown in the same conditions as previously described. *M. abscessus* was grown for biofilm assays in RPMI containing 2% heat-inactivated platelet poor plasma (HIPPP) for 4 days. Cultures were then adjusted to OD_650_ of 1.2, corresponding to 1×10^8^ cfu/ml.

A clinical strain of *S. aureus* was grown overnight in tryptic soy broth at 37°C with shaking. On the following day, fresh tryptic soy broth was inoculated with the overnight culture and grown for 4 hours prior to the experiment. *S. aureus* culture was then adjusted to an OD_650_ of 0.4, corresponding to 1×10^8^ cfu/ml. *Pseudomonas aeruginosa* strain PAO1 was grown overnight in complete RPMI at 37°C with shaking. PAO1 was then adjusted to an OD_650_ of 0.3, corresponding to 5×10^8^ cfu/ml. Samples of each bacterial preparation were plated to confirm correct titers.

*Neutrophil isolation*. Neutrophils were isolated from healthy volunteers by the plasma Percoll method as previously described [23]. Cells were confirmed to be >98% pure by visual inspection of cytospins, and resulting gene arrays had negligible expression of CD4 and CD8A, genes specific to peripheral blood mononuclear cells. Neutrophils were washed and resuspended in Krebs-Ringer phosphate-buffered saline with dextrose (KRPD; 154 mM NaCl, 5.6 mM KCl, 1.1 mM MgSO_4_, 2.2 mM CaCl, 0.85 mM NaH_2_PO_4_, 2.15 mM Na_2_HPO_4_ and 0.2% dextrose). These studies were approved by the National Jewish Health Institutional Review Board, and written informed consent approved by the National Jewish Health Institutional Review Board was obtained from all neutrophil donors. The study was conducted in accordance with the Declaration of Helsinki.

### Superoxide Release Assay

Superoxide anion release was assayed using the cytochrome *c* method [24]. Neutrophils (6×10^6^) were resuspended in KRPD supplemented with 2% HIPPP (complete KRPD) containing cytochrome *c*. Cells were stimulated with *M. abscessus* or *S. aureus* at an MOI of 10∶1 for all bacteria, or with neutrophils alone. Samples were pelleted at 15 and 60 minutes, supernatants were transferred to a 96-well plate, and read at an absorbance of 550 nm.

### Microarray Analysis of Neutrophil Gene Expression

Neutrophils (125×10^6^ per condition) were resuspended in RPMI with 2% HIPPP (complete RPMI), and treated with *M. abscessus* or *S. aureus* at an MOI of 10∶1. A control aliquot of neutrophils was treated with saline. All tubes were rotated end-over-end at 37°C. At 2 h, neutrophils were pelleted and RNA isolated using Trizol Reagent (Invitrogen). Microarray sample labeling was carried out using standard methods for reverse transcription and one round of *in vitro* transcription [25]. Affymetrix HG-U133A Plus 2.0 microarrays were hybridized with 10µg cRNA and processed per the manufacturer’s protocol at the University of Colorado Denver Genomics and Microarray core. Raw microarray data is available at Gene Expression Omnibus (Series record GSE39889).

Gene expression data were filtered for genes present in at least 3 samples. Expression data was further selected for those genes that changed significantly (P<0.01) as determined by ANOVA using MultiExperiment Viewer 4.6 [26]. Hierarchical clustering was performed using Euclidean distance and complete linkage. Venn diagrams were generated using BioVenn (http://www.cmbi.ru.nl/cdd/biovenn/) [27] for genes upregulated greater than or equal to 2-fold and less than 0.5-fold. For hierarchical clustering of cytokine and chemokine genes, genes belonging to the “cytokine activity” (GO:0005125) and “chemokine activity” (GO:0008009) GO categories from the Affymetrix analysis were used. Gene ontology analysis was performed by DAVID Bioinformatics Resources 6.7 (http://david.abcc.ncifcrf.gov/tools.jsp) [28] using “High” stringency to determine significantly regulated functional classes of genes.

### Cytokine and Chemokine Release

Secretion of cytokines and chemokines were determined after 2 h and 4 h stimulation with *M. abscessus* and *S. aureus* at a MOI of 10∶1. Products were quantified by ELISA for TNFα, IL8, CXCL2/GRO2, IL1β, CCL4/MIP1β, CCL20/MIP3α (ELISATech), IL1α, and CCL3/MIP1α (R&D Systems).

### Killing Assay

Neutrophils (5×10^6^) were suspended in complete RPMI. Sample tubes contained cells suspended in complete RPMI and the respective bacteria at a MOI of 10∶1. Control tubes contained each type of bacteria in complete RPMI. All tubes were incubated at 37°C with constant rotation at 8 rpm. At 2 and 4 hours an aliquot was removed to 0.01% Triton X-100. Following serial dilutions, *M. abscessus* was plated on 7H10 supplemented with OADC plates and *S. aureus* was plated on LB plates, and incubated at 37°C. Colonies were counted for *S. aureus* at 16–20 hours and for *M. abscessus* at 48–72 hours after plating.

### M. abscessus Phagocytosis

*M. abscessus* was washed, stained in PBS with 30 µg/ml FITC for 1 h at room temperature with constant agitation, and washed 3x with PBS. Neutrophils (1×10^6^) and *M. abscessus* were mixed at an MOI of 10∶1 for 1 and 2 hours. Samples were washed twice to remove free bacteria, and fixed in 1% formaldehyde/3% sucrose. Extracellular fluorescence was quenched with 0.1% Trypan Blue for 10 min, washed, and resuspended in PBS. Analysis was performed on a FacsCalibur flow cytometer (BD Biosciences) using FloJo software (TreeStar).

### Neutrophil Extracellular DNA Assay

Extracellular DNA associated with neutrophil extracellular trap (NET) formation was detected using a modification of the method of Fuchs *et al*. [29], as previously described [30]. Briefly, purified human neutrophils in complete RPMI were treated with each bacteria at an MOI of 2 at 37°C with rotation. At 4 hours for *M. abscessus* and 2 hours for *S. aureus*, limited nuclease digestion was performed with micrococcal nuclease (0.5 units/ml) for 10 minutes at 37°C. Nuclease activity was then stopped with 5 mM EDTA, and cellular debris was removed by centrifugation. DNA content was measured with the Quant-iT™ Picogreen assay (Invitrogen).

### Neutrophil Death Analysis

Neutrophils in complete RPMI were stimulated with *M. abscessus* or *S. aureus* at an MOI of 10∶1 for 4 hours at 37°C with constant rotation at 8 rpm. As a control, neutrophils were also incubated in the same conditions solely with complete RPMI. Neutrophil necrosis was determined by the percent of total lactate dehydrogenase release using the Cytotoxicity Detection Kit (Roche) [31]. Apoptosis was quantified by immunoassay for cytoplasmic histone-associated DNA fragments (Roche) and reported as the percent of histone-bound DNA in a given sample relative to the level of histone-bound DNA detected in neutrophils in which apoptosis was induced by cycloheximide [31]; after cell lysis, this assay uniquely identifies soluble chromatin fragments that are characteristic of apoptotic cells.

### Biofilm Density Assay

*M. abscessus* grown in complete RPMI was added at an MOI of 10∶1 to 2.4×10^6^ neutrophils per well (8 wells per condition) in complete RPMI in a round-bottom 96-well plate (Nunc) as previously described [32]. A screening assembly (Transferable Solid Phase Screening System, Nunc), composed of a non-coated polystyrene 96-well plate lid with pegs that extend into each well, was placed on the 96-well plate, and incubated for 5 days with rocking (three to four oscillations per minute) at 37°C. Over this time, neutrophil death is complete, and any killing activity by neutrophils is presumably lost. The peg assembly was stained in 1% crystal violet and readings were obtained in a plate reader at 550 nm (KCjunior software; BioTek Instruments). Control plates contained bacteria alone, and neutrophils alone, which caused minimal crystal violet staining. Values were averaged before comparison of each test well.

### Biofilm Growth in Flow Chambers

Neutrophils (4.8×10^6^) were suspended in complete RPMI and allowed to adhere to a glass coverslip secured to a 3-chamber flow cell (3×1.5×45 mm). *M. abscessus* was added simultaneously with neutrophils and allowed to adhere for 2 h before flow was initiated at 3 ml/hour/channel for 5 days. Flow chambers were stained with Live/Dead stain (0.5 µM Syto9 and10 µM propidium iodide) and imaged *in situ*.

### Statistical Analysis

Data was analyzed by one-way ANOVA with Bonferroni post-test, unless otherwise noted. Significance was set at a p-value of 0.05.

## Results

### M. abscessus Promotes Superoxide Anion Release

**Figure 1:**
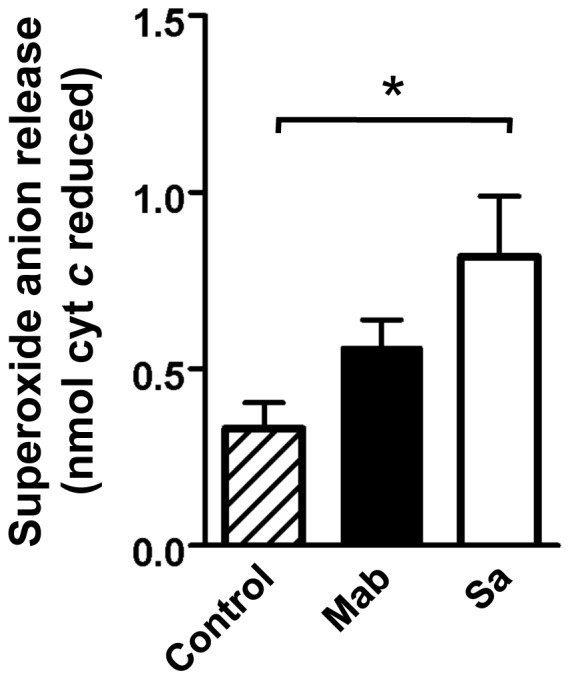
Superoxide anion release in response to *M. abscessus*. *M. abscessus* (Mab; *closed bar*), or *S. aureus* (Sa; *open bar*) were added to neutrophils at a 10∶1 MOI, and superoxide release was compared to control neutrophils (*hatched bars*) by measuring reduction of cytochrome *c* after 60 min. Data is presented as mean±SEM; Bonferroni post-test indicated significant release of superoxide; n = 10, *p<0.05.

Superoxide anion formation is central to the bactericidal capacity of neutrophils, but also implicated in the injurious effect of overexuberant inflammation. *S. aureus* was chosen as a positive control for its known ability to induce superoxide anion release, and for its predominance in both the CF airway and in soft tissue abscesses. *M. abscessus* consistently induced superoxide anion release after 60 min (**Fig. 1**), although this release was not significantly greater than control cells by multiple comparison by ANOVA. However, *S. aureus* released significant amounts of superoxide anion. Superoxide anion release over baseline was not observed at 15 min by either bacteria (data not shown).

### M. abscessus Gene Expression Signatures

**Figure 2:**
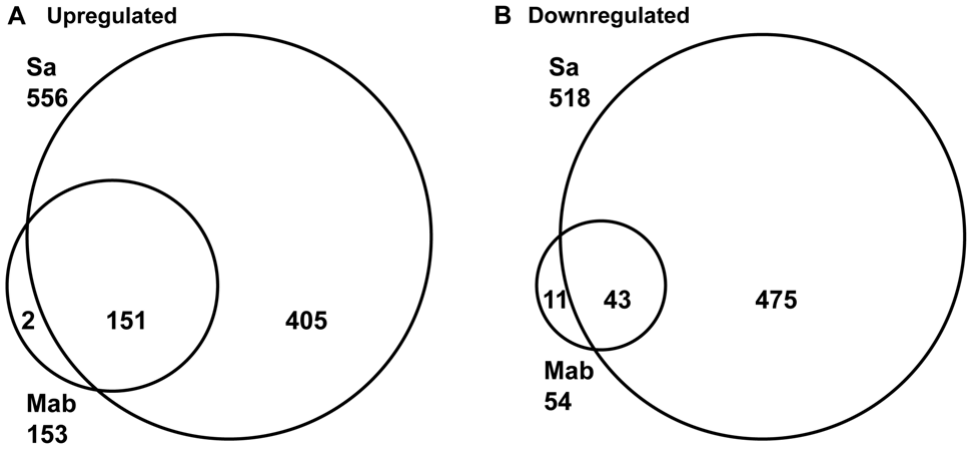
Gene expression analysis indicates a core of commonly induced genes. **A**, Total number of unique upregulated genes by *M. abscessus* (Mab), and *S. aureus* (Sa) are indicated near the organism name, and overlapping genes are indicated within sections of the Venn diagram. **B**, Downregulated genes. Analysis was performed on 4 independent experiments.

To broadly define neutrophil response to *M. abscessus,* global gene expression patterns were analyzed. Following 2 h exposure of neutrophils to *M. abscessus* or *S. aureus*, a total of 153 and 556 unique genes, respectively, were significantly upregulated greater than two-fold (**Fig. 2A** and **Table 1**). The majority of genes expressed by *M. abscessus* were commonly expressed with neutrophils stimulated by *S. aureus*, although *S. aureus* induced a more robust response (**Table 1**). Hierarchical clustering of all significantly expressed genes demonstrated three major clusters consisting of control, *M. abscessus*-stimulated, and *S. aureus*-stimulated neutrophils (data not shown). Therefore, *M. abscessus* elicits limited gene expression in neutrophils, and a core gene expression profile exists for neutrophils stimulated with these bacteria.

**Table 1:** Genes most upregulated by *M. abscessus*.

Gene Symbol	*M. abscessus* fold-change^1^	*S. aureus* fold-change	ID^2^
IL1A	72.8	476.3	210118_s_at
CCL20	33.0	330.1	205476_at
CXCL2	19.0	222.8	1569203_at
EST^3^	18.2	228.8	237204_at
IL6	16.7	718.5	205207_at
BIC	14.2	85.8	229437_at
miRNA-146A	11.5	20.5	232504_at
EST	11.4	110.0	236982_at
CXCL3	10.2	100.5	207850_at
C15orf48	8.4	53.6	223484_at
TNF	7.8	27.7	207113_s_at
CCL3	7.3	14.4	205114_s_at
CCL2	7.3	22.7	216598_s_at
DUSP2	7.2	32.1	204794_at
KMO	6.9	23.8	211138_s_at
TRAF1	6.5	33.7	205599_at
INA	6.2	8.8	204465_s_at
EST	6.1	41.9	235739_at
EST	6.0	7.5	240231_at
IL1B	6.0	13.3	39402_at

1 Fold-change compared to non-stimulated neutrophils.

2 Affymetrix Probe Set ID.

3 Expressed sequence tag.

Cytokines and chemokines were among the top genes induced by each bacterium (**Table 1**). The importance of chemokine gene expression was confirmed by gene ontology analysis, which identified a set of chemokines as the lone significant functional gene class regulated by *M. abscessus*; genes in this set include *CXCL2*, *CXCL3*, *CCL4*, and *CCL20*. Genes for *IL1A*, *CCL20*, *CXCL2*, *CXCL3*, *TNF*, and *IL6* were highly and commonly induced by each bacterium. Other genes induced by both bacteria were three ESTs (represented by probes 237204_at, 235739_at, and 236982_at), *C15orf48*, and *DUSP2*. In addition, *CCL2*, *CCL3*, *KMO*, and an EST (232504_at) were among the most induced by *M. abscessus*. Interestingly, two miRNA genes, *BIC* (miRNA-155) and 232504_at (miRNA-146A) were detected. *S. aureus* strongly induced a number of genes that were minimally induced by *M. abscessus* including *NSUN6*, *IL23A*, *GPR4*, *FOSL1*, *NR1D1*, and *ERN1* (data not shown).

Neutrophils stimulated with *M. abscessus* and *S. aureus* showed downregulation of a number of genes (**Fig. 2B**). Several shared downregulated genes were evident, although *S. aureus* reduced more genes than *M. abscessus*. The genes shared within the 20 most downregulated genes by both stimuli included *CCR2*, *FAM26B*, *PCCB*, and *MPZL1* (data not shown).

**Figure 3:**
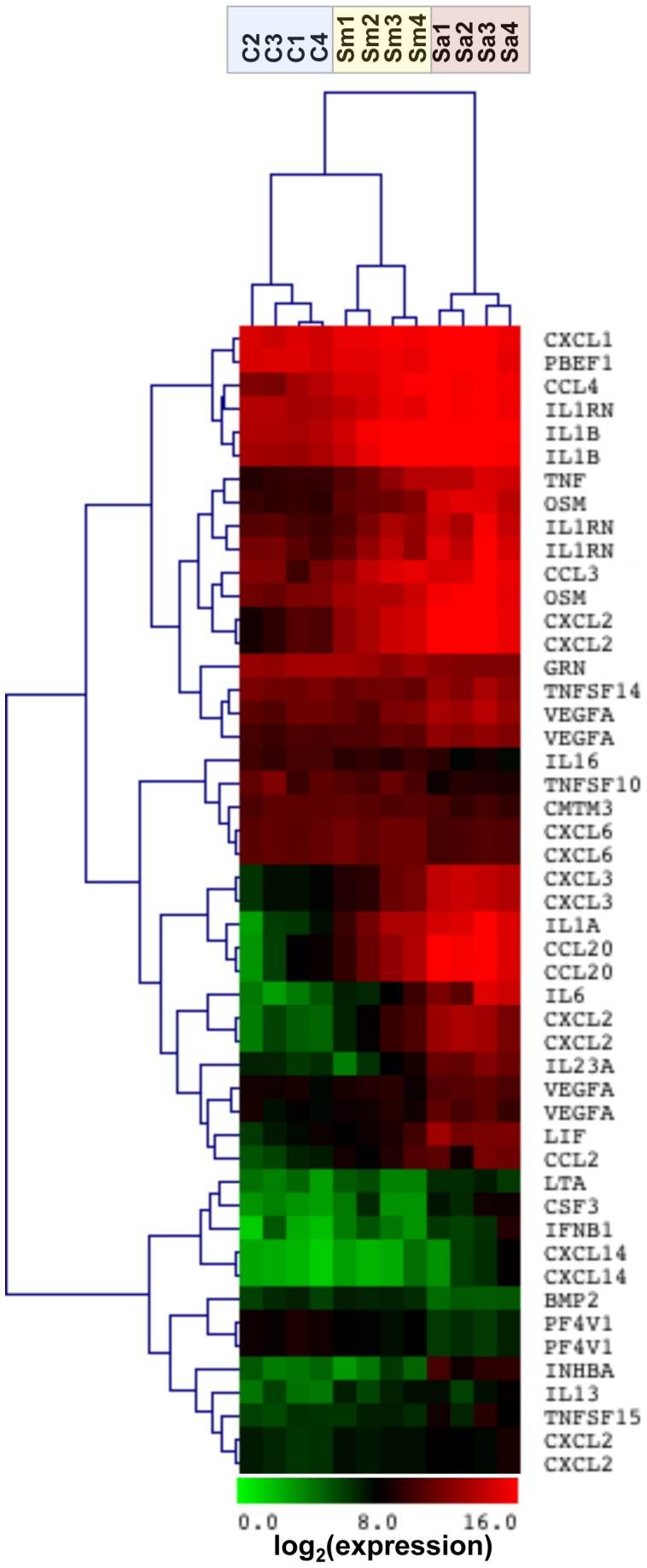
Hierarchical clustering of cytokine and chemokine genes by *M. abscessus*. Neutrophils were stimulated with *M. abscessus* (Sm; *yellow shading*), or *S. aureus* (Sa; *red shading*) for 2 hours, and gene expression was determined compared to non-stimulated neutrophils (C; *blue shading*). Tree spacing indicates linkage distance. Highly expressed (*red*) and low expressed (*green*) genes are indicated from 4 different neutrophil donors.

To better understand cytokine and chemokine expression we compared significantly changed genes via hierarchical clustering defined by the GO categories “cytokine activity” and “chemokine activity”. Based on cytokine and chemokine expression, *M. abscessus* clustered together and uniquely from both control and *S. aureus*-stimulated neutrophils (**Fig. 3**). *M. abscessus* induced genes for neutrophil chemoattractants (CXCL2, CXCL3) and macrophage inflammatory proteins (MIPs), including CCL2, CCL3, CCL4, and CCL20. In addition to neutrophil chemoattractant genes (*CXCL1*, *CXCL2*, *CXCL3*), *S. aureus* strongly induced genes for proinflammatory (*TNF*, *IL1A*, *IL1B*, *IL6*) and immunomodulatory (*OSM*, *IL23A*, *IL1RN*, *LIF*, *VEGFA*) proteins; in all cases the gene induction by *S. aureus* was greater than that of *M. abscessus*. Cytokine genes downregulated only by *S. aureus* include *TNFSF10*/*TRAIL* and *CXCL6* (data not shown). Therefore, the cytokine and chemokine induction pattern for *M. abscessus* is similar, but weaker than that of *S. aureus*, and enriched in neutrophil chemoattractants and MIP genes.

**Figure 4:**
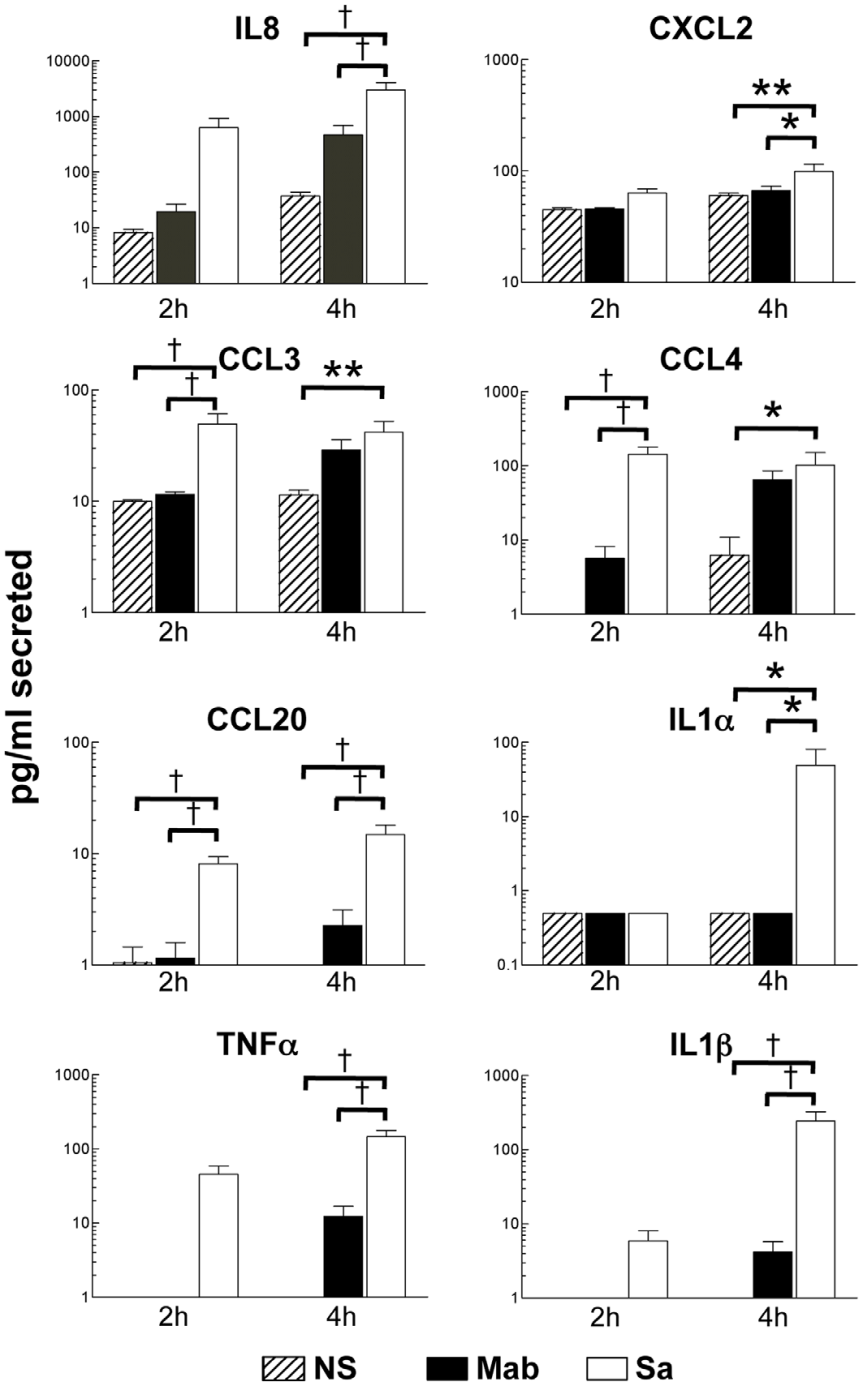
Secretion of chemokines and cytokines. Neutrophils were stimulated with *M. abscessus* (Mab; *closed bars*), or *S. aureus* (Sa; *open bars*) for 2 and 4 hours, or left unstimulated (NS; *hatched bars*) and supernatants were collected. The indicated cytokine and chemokine levels were determined by ELISA. Mean±SEM, n = 7–8; *p<0.05; **p<0.01; †p<0.001 by Bonferroni post-test analysis.

As predicted from the gene expression data, cytokine and chemokine secretion was observed by both *M. abscessus* and *S. aureus* (**Fig. 4**). However, the secretion of all cytokines tested was consistently and significantly greater for *S. aureus* (by Bonferroni post-test analysis of two-way ANOVA, which confirmed an effect of bacteria, p<0.01 for all treatments). Cytokine secretion, with the exception of CXCL2 and IL1α, was significantly enhanced in the presence of *M. abscessus* after 4 hours of exposure when compared directly to unstimulated cells (data not shown and **Fig. 4**; statistical analysis by t-test). CCL4 alone was significantly secreted by stimulation at 2 hours exposure to *M. abscessus* when compared only to unstimulated neutrophils. In contrast, *S. aureus* induced significant secretion of all cytokines at this earlier time, with the exception of CXCL2 and IL1α, and at 4h of *S. aureus* treatment. Therefore, *S. aureus* induces a more robust and rapid cytokine response than *M. abscessus*.

### Neutrophil Killing of Bacteria

**Figure 5:**
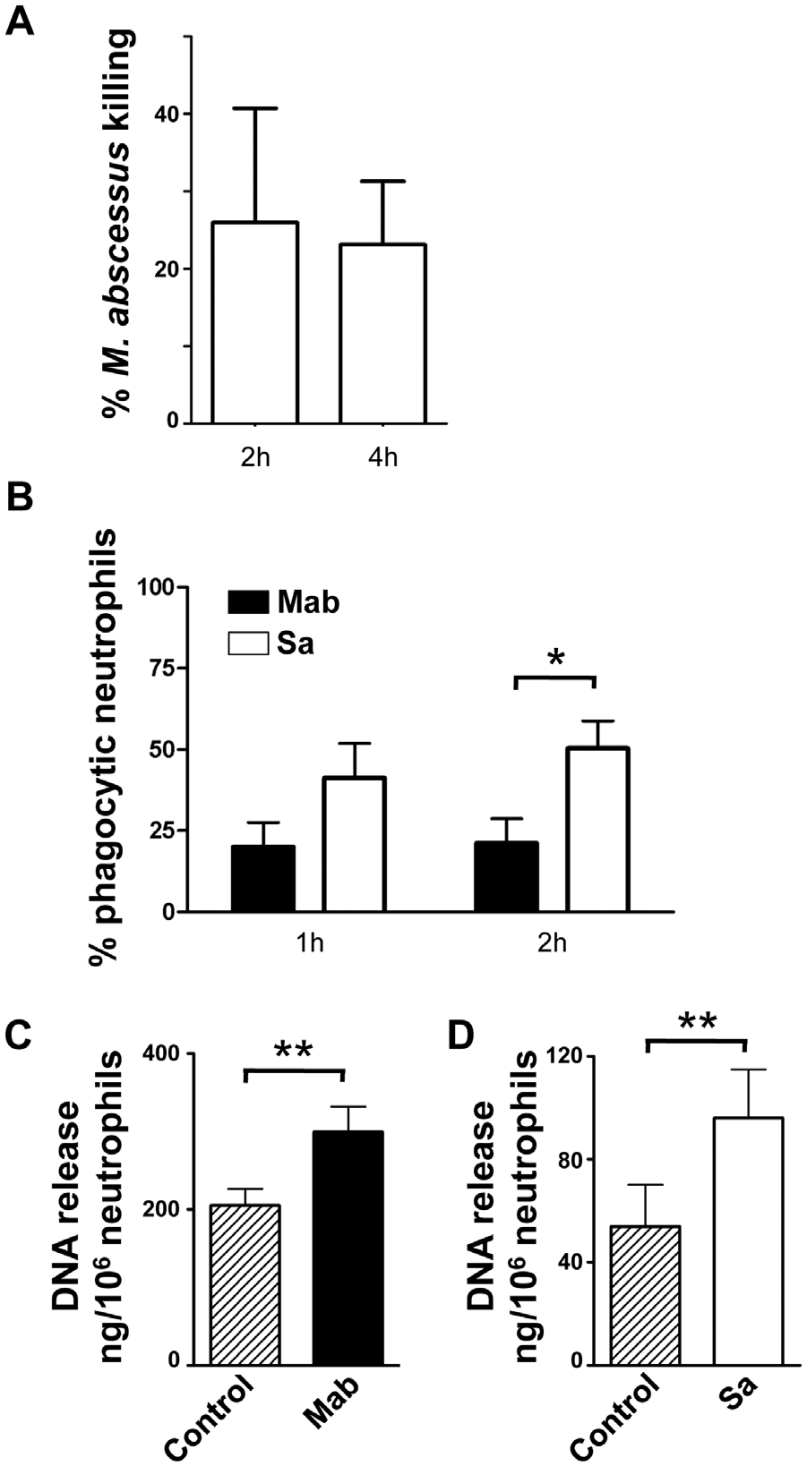
Killing and phagocytosis of *M. abscessus*. **A**, Neutrophil killing of *M. abscessus*. Neutrophils were incubated with *M. abscessus* for 2 and 4 hours; surviving bacteria were enumerated and compared to bacterial cfu in the absence of neutrophils for the same time points to obtain percent killing. Data represents mean±SEM of 6 experiments. **B**, Phagocytosis of *M. abscessus* by neutrophils. FITC-labeled bacteria were incubated with neutrophils for the indicated times, and the percent of neutrophils with intracellular staining was determined. Data represents mean±SEM of 10 experiments. Two-way ANOVA was performed; *p<0.05 by Bonferroni post-test. **C**, Extracellular DNA release. Neutrophils were exposed to *M. abscessus* (Mab) at a MOI of 2 for 4 hours. Data represents mean±SEM; n = 13. **D**, Extracellular DNA release by neutrophils exposed to *S. aureus* (Sa) at an MOI of 2 for 2 hours. Data represents mean±SEM; n = 6. Analysis for significance for **C** and **D** was determined by a paired, one-tail t test.

The abundance of neutrophils at the site of *M. abscessus* infection suggests that neutrophils are relatively ineffective at controlling *M. abscessus* infections. The ability of neutrophils to kill *M. abscessus* was tested *in vitro*. Neutrophils are capable of killing *M. abscessus* as early as 2 hours after exposure (**Fig. 5A**). Under the conditions studied approximately 25% of *M. abscessus* was killed, compared to 80% of *S. aureus*, although direct comparison is complicated by the different times and conditions for optimal killing, different growth rates of each bacteria, and different rates of neutrophil survival. To more directly compare killing mechanisms, we measured phagocytosis. While neutrophils effectively phagocytosed *M. abscessus* at both 1 and 2 hours (**Fig. 5B**), phagocytosis was greater for *S. aureus*. Neutrophil extracellular trap (NET) formation is also implicated in the control of infections. *M. abscessus* induced the release of DNA from neutrophils to a similar extent as *S. aureus* (**Fig. 5C, D**). These data suggest that killing of *M. abscessus* involves both phagocytosis and NETs.

### Neutrophil Death by Necrosis and Apoptosis

**Figure 6:**
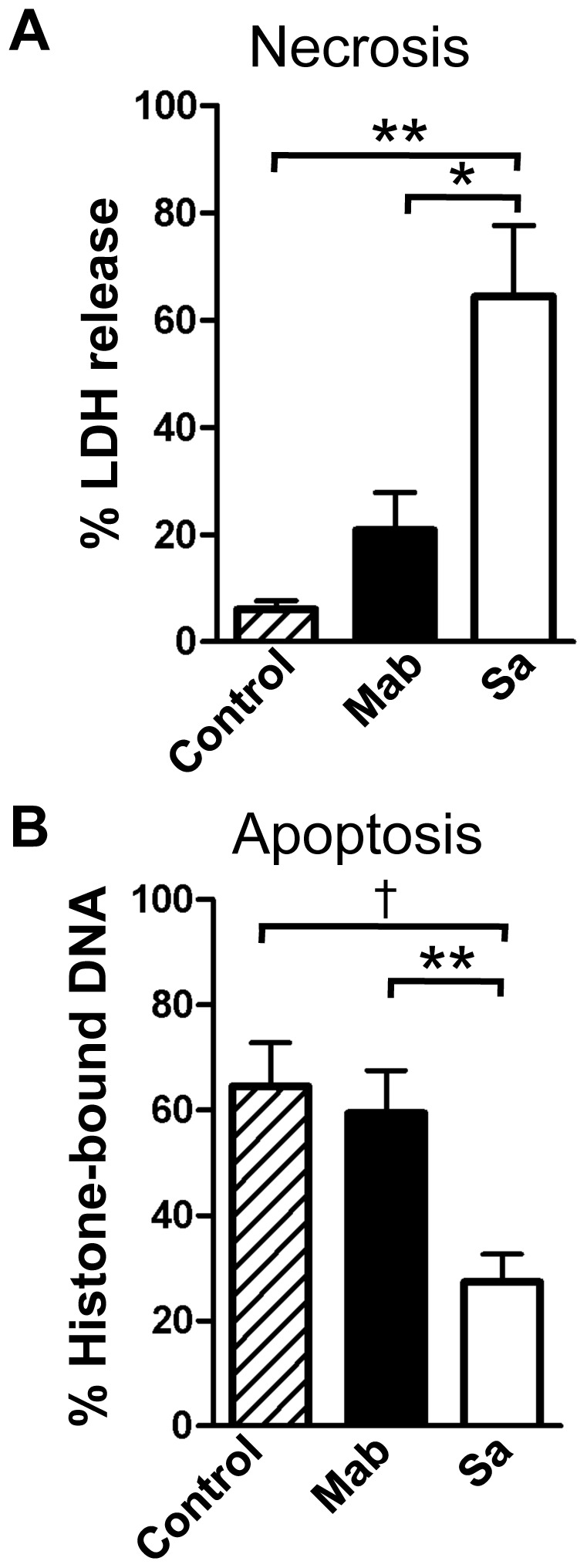
*M. abscessus* does not alter neutrophil death pathways. Neutrophils were incubated with *M. abscessus* (Mab; *closed bars*) or *S. aureus* (Sa; *open bars*) for 4 hours. **A**, Necrotic cell death was measured by release of lactate dehydrogenase (LDH). **B**, Apoptotic cell death was measured by solubilized histone-bound DNA. Only *S. aureus* significantly changed either necrosis or apoptosis compared to non-stimulated neutrophils. Data represents mean±SEM; n = 6–9; *p<0.05, **p<0.01, †p<0.001 by Bonferroni post-test analysis.

Because *M. abscessus* survives for long periods of time in neutrophil-rich abscesses, we explored the possibility that changes in cell death contribute to neutrophil accumulation. Necrotic neutrophil death in the presence of *M. abscessus* was not different from that of untreated cells (**Fig. 6A**), whereas *S. aureus* induced significant necrotic cell death when compared to either control neutrophils or *M. abscessus*-treated neutrophils. Similarly, apoptosis of neutrophils exposed to *M. abscessus* was not different than spontaneous apoptosis in unstimulated neutrophils, whereas *S. aureus* inhibited apoptosis when compared to either condition (**Fig. 6B**). Results were similar when cell death was measured by 7-AAD, a fluorescent DNA binding dye that detects necrotic and apoptotic cells (data not shown). Therefore, under the conditions tested, cell death pathways are not greatly changed in the presence of *M. abscessus* compared to unstimulated neutrophils, whereas *S. aureus* induces necrotic cell death.

### Neutrophil-induced Enhancement of M. abscessus Biofilm Formation

**Figure 7:**
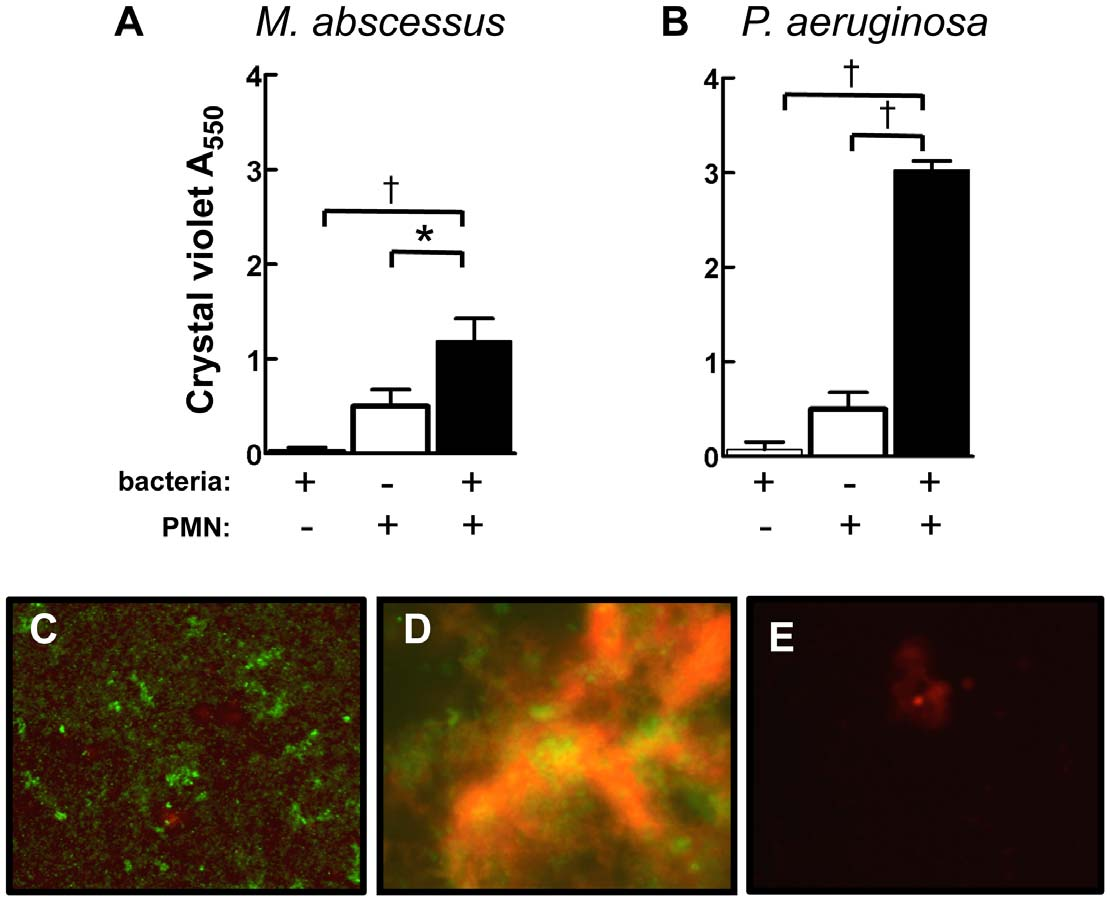
*M. abscessus* biofilm density is enhanced by neutrophils. Bacteria (*hatched bars*), neutrophils (*open bars*), or the combination of bacteria and neutrophils (*closed bars*) were incubated for 5 days, and biofilm density was assessed by crystal violet staining in response to **A**, *M. abscessus*; **B**, *P. aeruginosa*. Data represent mean±SEM from 4–9 experiments. *p<0.05; †, p<0.001. Representative biofilm formation in a flow chamber by: **C**, *M. abscessus* alone (10×); **D**, *M. abscessus* and neutrophils (40×); **E**, neutrophils alone (40×). Biofilms were stained for live (*green*) and dead (*red*) cells.

Compared to planktonic bacteria, biofilms confer a reduced host response and increased antibiotic resistance. Failure of host defense to eradicate *M. abscessus*, combined with poor response to antibiotic treatment [17], suggests that biofilm formation may contribute to its pathogenesis. Dead and dying neutrophils enhance *P. aeruginosa* biofilm formation [32]–[34], but this effect has not been described for *M. abscessus*. Although some strains of the smooth morphotype of *M. abscessus* readily form biofilms [18], in a static biofilm plate *M. abscessus* formed relatively scant biofilms by 72 hrs (**Fig.** **7A**), or after 4 weeks of culture in complete RPMI, 7H9/ADC, or 7H9 alone (data not shown). However, the presence of necrotic neutrophils significantly augmented *M. abscessus* biofilm formation (**Fig.** **7A**). When compared to *M. abscessus*, *P. aeruginosa* formed equally poor biofilms in the presence of bacteria alone; however, neutrophils greatly enhanced biofilm formation by *P. aeruginosa* (**Fig. 7B**). Biofilm formation in the presence of *P. aeruginosa* and neutrophils was significantly greater than those formed in the presence of *M. abscessus* and neutrophils (P<0.001 by Bonferroni post-test comparison of two-way ANOVA). Under dynamic conditions using a flow chamber reactor, we observed biofilm formation with *M. abscessus* alone (**Fig. 7C**), and biofilm formation was consistently enhanced in the presence of necrotic neutrophils (**Fig. 7D**). Neutrophils alone provided little staining (**Fig. 7E**). Therefore, these data suggest that the ability of *M. abscessus* to form biofilms is enhanced in an environment that mimics neutrophil-rich abscesses and the CF airway, and that this may contribute to its virulence and purulence.

## Discussion

Large numbers of neutrophils are nearly always present during, and often prior to, infection with *M. abscessus* [1], [4], [9]–[11], [13], [35]–[37] and are uniformly abundant when the pathogen infects the airways of patients with CF or other forms of bronchiectasis [38]–[40]. Initial infection occurs from environmental strains of *M. abscessu*s, which are predominately smooth variants, so the effect of smooth *M. abscessus* on neutrophil functions is important in understanding disease pathogenesis. Little is known concerning the initial host response to *M. abscessus*, where diagnosis may lag the initial infection by months or years. In experimental models of *M. abscessus* the presence of increased numbers of neutrophils was associated with worse response to the infection [41]. Based on these observations, we hypothesize that the neutrophil response to the smooth variant of *M. abscessus* is largely ineffective and may instead create a favorable environment for the bacteria that is central to the pathogenesis of infection, leading to perpetuation of inflammation and a survival advantage for the bacterium.

Data presented herein suggest that many features of *M. abscessus* infection can be attributed to the response of human neutrophils. *M. abscessus* contributes to neutrophil accumulation to the site of infection through directed release of IL8 by macrophages and epithelial cells [16], [42], [43]. In addition, neutrophils may be involved in a positive feedback loop by also producing the neutrophil chemoattracants CXCL3 and IL8. These findings are consistent with the neutrophil-rich environment observed in *M. abscessus* infections.

The initial neutrophil response includes superoxide anion release, which may contribute to *M. abscessus* killing. Susceptibility to superoxide anion is highly variable between bacterial species, as *S. aureus* is highly sensitive, while *P. aeruginosa* killing occurs largely independent of superoxide. In the case of *M. abscessus*, both superoxide anion release and neutrophil killing is limited, and suggests that *M. abscessus* possesses mechanisms for avoiding reactive oxygen species accumulation and/or superoxide-dependent death, such as expression of superoxide dismutase and catalase, as seen with *M. tuberculosis* [44]. In endothelial cells, *M. abscessus* produced relatively little reactive oxygen species, yet was a relatively strong inducer of reactive nitrogen species compared to other mycobacteria [15]. Recently, oxidative stress in macrophages was shown to be important in supporting the growth of *M. abscessus* rather than as a killing mechanism [43]. In addition, superoxide release may lead to local inflammation and tissue damage, resulting in additional neutrophil recruitment to the site of infection. While greater study of the effects of reactive oxygen species on *M. abscessus* killing is required, we have shown that phagocytosis may be an important component of killing. When compared to *S. aureus*, *M. abscessus* displayed less phagocytosis. This observation may also explain the lower levels of superoxide anion release, as phagolysosome formation may promote formation of reactive oxygen species. However, *M. abscessus* was able to release significant amounts of extracellular DNA, suggesting that NETs are also involved in bacterial clearance. Therefore, when compared to *S. aureus*, a pathogen relevant for its presence in abscesses, *M. abscessus* demonstrates less robust superoxide anion release and phagocytosis, which may contribute to survival of *M. abscessus* in neutrophil-rich environments.

Reduced immune recognition and killing described above may support growth and persistence of *M. abscessus*. Concurrently, neutrophil cell death pathways are dysregulated by *M. abscessus*; neutrophil necrotic death is limited, and inhibition of spontaneous apoptosis does not occur. In contrast, *S. aureus* stimulates significant necrotic cell death and inhibits apoptosis. In the case of *S. aureus*, this pattern of cell death suggests an infection environment that overall is enriched in cell fragments and pro-inflammatory material. On the other hand, the lack of significant necrosis suggests that *M. abscessus* infection may occur in the absence of neutrophil-derived inflammatory materials; however, apoptotic death could promote either additional pro-inflammatory or anti-inflammatory signals depending on the efficiency of apoptotic cell clearance. During chronic infections involving *M. abscessus*, the presence of neutrophils that demonstrate post-apoptotic necrosis, as well as low levels of primary necrosis, may lead to considerable accumulation of cellular debris.

Two mechanisms that maintain survival and determine the pathogenic potential of *M. abscessus* include the extensive reorganization of the cell surface (smooth to rough morphotype) and biofilm formation [17]–[19]. These survival mechanisms resemble those successfully employed by *P. aeruginosa*, another opportunistic pathogen that can thrive in the neutrophil-rich CF airway through cell wall adaptation (mucoidy) and biofilm formation [45]. We have studied the predominant environmental smooth morphotype to better model initial infection. Previously, we have reported that P. aeruginosa exploits neutrophil-derived polymers of F-actin and DNA to serve as a scaffold that greatly enhances early biofilm formation 3234. Smooth variants of M. abscessus 18 and of other species of nontuberculous mycobacteria 4648 have been identified as having a propensity to form biofilms, although Williams et al. 49 demonstrated biofilm formation by both M. abscessus morphotypes. The presence of neutrophils resulted in robust *M. abscessus* biofilm development, in both a flow cell reactor and a static biofilm plate (Fig. 7). While biofilm formation may depend on growth conditions 18, substrates, and M. abscessus strains, these data suggest that M. abscessus is capable of expressing a more virulent phenotype in the presence of neutrophils. In contrast to an earlier report that biofilm formation by *M. abscessus* occurred over a period of weeks [50], we found that in the presence of neutrophils, M. abscessus biofilms could form quickly in conditions more closely resembling the nutrient rich CF airway or inflamed tissue. These data suggest that initial and established infections may exploit the neutrophil-rich environment to maintain an infectious state.

In addition to chemokines, proinflammatory cytokines were produced by neutrophils, including TNFα and IL1ß. This is consistent with data using human peripheral blood mononuclear cells [51]. However, infection of macrophages by smooth *M. abscessus* resulted in minimal release of TNFα [14], [52], [53]. In macrophages, expression of GPL by smooth *M. abscessus* masks recognition of cell wall phosphatidyl-*myo*-inositol mannosides [53], and GPL loss in rough *M. abscessus* is also associated with increased synthesis of cell surface lipoproteins [52]. In macrophages, pro-inflammatory response to both *M. abscessus-*derived phosphatidyl-*myo*-inositol mannosides and cell surface lipoproteins occurs predominately through Toll-like receptor 2 [52]–[54] with the involvement of dectin-1 and the NLRP3 inflammasome [42], [54]. Our data suggests that the smooth variant is able to activate a cytokine response in neutrophils, although the receptors involved and comparison to the rough variant awaits further studies. In addition, the activation of neutrophils by the smooth variant further indicates that neutrophil responses are important in initial host response. Interestingly, the secretion of IL1α and IL1ß by *M. abscessus* in neutrophils was minimal; thus, the extensive induction of genes for these cytokines suggests a translational deficit or the inability to activate inflammasomes. Therefore, mechanisms of activation by *M. abscessus* appear to be cell-specific. Compared to *S. aureus*, cytokine secretion in response to *M. abscessus* is reduced and delayed. Therefore, a primary neutrophil response to *M. abscessus* indicates a pro-inflammatory environment and reduced immune surveillance that is both temporally and quantitatively limited. These findings are consistent with a host response that results in a persistent but rather indolent infection, which is often described with *M. abscessus* in the clinical setting.

To our knowledge, this study is the first describing the neutrophil response to *M. abscessus*. Data presented herein suggest that neutrophil-*M. abscessus* interactions may explain many features of *M. abscessus* infection. *M. abscessus* induces neutrophil accumulation to the site of infection through selective transcription and translation of chemokines by the neutrophil and other host cells [42], [43]. Neutrophils respond with superoxide anion release, which may contribute to local inflammation and tissue damage, and may enhance survival of the pathogen [43]. Infection and persistent accumulation of leukocytes may result in an environment rich in cellular debris, which our data suggests the pathogen exploits to accelerate biofilm formation. The biofilm phenotype evokes greater resistance to both antimicrobials and immune control, likely contributing to the low success rate of medical treatment of this infection. The pattern of neutrophil activation by *M. abscessus* is in contrast to that of *S. aureus*, another pathogen that also is maintained in neutrophil-rich environments, in that *S. aureus* strongly and rapidly stimulates neutrophil responses. These data suggest two approaches to virulence: one that promotes rapid and extensive inflammation that may act to overwhelm immune responses, and one that exploits a subdued immune response that may allow stealth bacterial growth and maintenance of infection. In this way, *M. abscessus* may promote a neutrophil-dependent growth niche, and suggests potential therapeutic approaches to promote clearance of *M. abscessus*.
